# Corticospinal fibers with different origins impair in amyotrophic lateral sclerosis: A neurite orientation dispersion and density imaging study

**DOI:** 10.1111/cns.14270

**Published:** 2023-05-19

**Authors:** Nao‐Xin Huang, Wen Qin, Jia‐Hui Lin, Qiu‐Yi Dong, Hua‐Jun Chen

**Affiliations:** ^1^ Department of Radiology Fujian Medical University Union Hospital Fuzhou China; ^2^ Department of Radiology and Tianjin Key Laboratory of Functional Imaging Tianjin Medical University General Hospital Tianjin China

**Keywords:** amyotrophic lateral sclerosis, corticospinal tract, diffusion tensor imaging, fiber tractography, neurite orientation dispersion and density imaging

## Abstract

**Aims:**

To investigate microstructural impairments of corticospinal tracts (CSTs) with different origins in amyotrophic lateral sclerosis (ALS) using neurite orientation dispersion and density imaging (NODDI).

**Methods:**

Diffusion‐weighted imaging data acquired from 39 patients with ALS and 50 controls were used to estimate NODDI and diffusion tensor imaging (DTI) models. Fine maps of CST subfibers originating from the primary motor area (M1), premotor cortex, primary sensory area, and supplementary motor area (SMA) were segmented. NODDI metrics (neurite density index [NDI] and orientation dispersion index [ODI]) and DTI metrics (fractional anisotropy [FA] and mean/axial/radial diffusivity [MD/AD/RD]) were computed.

**Results:**

The patients with ALS showed microstructural impairments (reflected by NDI, ODI, and FA reductions and MD, AD, and RD increases) in CST subfibers, especially in M1 fibers, which correlated with disease severity. Compared with other diffusion metrics, NDI yielded a higher effect size and detected the greatest extent of CST subfibers damage. Logistic regression analyses based on NDI in M1 subfiber yielded the best diagnostic performance compared with other subfibers and the whole CST.

**Conclusions:**

Microstructural impairment of CST subfibers (especially those originating from M1) is the key feature of ALS. The combination of NODDI and CST subfibers analysis may improve diagnosing performance for ALS.

## Graphical abstract

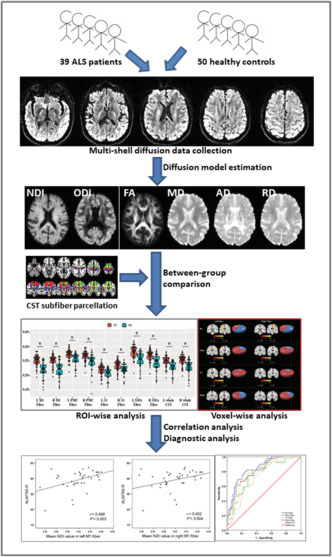

## 1 INTRODUCTION

Amyotrophic lateral sclerosis (ALS) is a fatal neurodegenerative disease that primarily involves the upper and lower motor neurons. Its pathological hallmarks are combined degeneration of the corticospinal tract (CST) and anterior horn cells, resulting in a decline in motor functions.^1^, ^2^ On average, its diagnosis is delayed 9–16 months due to the inadequate sensitivity of clinical examinations.^3^ Furthermore, most ALS patients die of respiratory dysfunction within 3–5 years of diagnosis.^3^ A consensus has been reached that the timely diagnosis of ALS is crucial for early intervention and prognosis.^4^ As such, it is crucial to adequately illuminate the brain structural and functional alterations related to ALS, which could help in identifying new neuroimaging biomarkers for diagnosis and monitoring disease progression.

Magnetic resonance imaging (MRI) is considered the leading tool for biomarker identification in ALS.^5^, ^6^ Neuroimaging studies using MRI have revealed macrostructural and microstructural impairments of the brain in ALS. For instance, structural MRI studies on ALS have confirmed that widespread gray matter alterations, including atrophy in several cortical and subcortical regions (such as the medial frontal gyrus, cingulate gyrus, caudate head, and thalamus)^7^ and decreased complexity in several cortical areas (such as the precentral and postcentral gyrus, cingulate gyrus, and circular sulcus of insula).^8^ White matter (WM) impairments in ALS have also been well documented^9^, ^10^, ^11^, ^12^: while diffusion tensor imaging (DTI) investigations have revealed impaired microstructural integrity in the corona radiata, posterior limb of the internal capsule, corpus callosum, and cingulate gyrus of ALS patients, diffusion kurtosis imaging (DKI) studies have documented decreased microstructural complexity in the CST pathway, corpus callosum, and precentral gyrus.

However, confidence in using the conventional DTI technique to complete the task of detecting WM impairment is limited because of low‐spatial resolution and an inability to represent the crossing of multiple fibers.^13^ Furthermore, DTI and DKI both adopt the “signal representations” approach, which lacks specificity and allows only an indirect characterization of microstructures.^14^ To avoid the abovementioned drawbacks, another advanced multi‐compartment diffusion model known as “neurite orientation dispersion and density imaging (NODDI)”^15^, ^16^ that can facilitate the estimation of the microstructure of dendrites and axons in the brain^17^ was proposed recently. Based on a “tissue model”,^18^ this technique enables the estimation of biologically relevant parameters, including neurite density index (NDI) and orientation dispersion index (ODI), which estimate the intracellular volume fraction and the degree of fiber coherence, respectively,^17^, ^19^ and the biological relevance of NODDI parameters has already been demonstrated in histology studies of animal and human brains.^20^ The NODDI approach has already been adopted for detecting WM microstructural abnormalities in various neurodegenerative diseases, such as Wilson's disease,^21^ Alzheimer's disease,^22^ and multiple sclerosis.^23^ Prior studies have consistently provided evidence that NODDI is more persuasive than conventional DTI in clinical studies.^20^ In patients with ALS, an NODDI study revealed NDI reductions in extensive regions, including the CST pathway, the corpus callosum, and the right precentral gyrus, together with ODI reductions in the right anterior internal capsule and precentral gyrus.^24^

Although existing diffusion MRI (dMRI) studies based on various models (such as DTI, DKI, and NODDI) have consistently reported that one of the classic WM pathologies of ALS is CST degeneration,^2^, ^25^ this result is confounded by the fact that CST subfibers have different origins. Specifically, although the primary motor area (M1) is considered to be the primary origin of CST fiber, some CST subfibers also originate from the premotor cortex (PMC), supplementary motor area (SMA), and primary somatosensory area (S1).^26^, ^27^ According to a previous diffusion study, the characteristics of CST subfibers with different origins, such as their tract volumes, directionality, and diffusivity, are distinctive.^28^ Moreover, differences in the function of CST subfibers according to their cerebral origin have been reported^29^, ^30^: M1 is responsible for the execution of movement, the PMC is mainly involved in visually guided movements, the SMA is used to plan and coordinate internally generated movements, and S1 is mainly concerned with descending control of sensory afferent input generated by movements. As recent studies have suggested, fully and accurately identifying the impairments in distinct CST subfibers caused by neurological disorders is indispensable, as it can, in turn, contribute to identifying biomarkers with greater sensitivity to facilitate earlier diagnosis and decision‐making regarding more aggressive treatment. For example, in a study on stroke,^31^ the impairment of CST subfibers originating from the M1 and the SMA (rather than the PMC and S1) was closely associated with the motor outcomes of patients, suggesting that recognizing impairments in these subfibers is crucial for assessing the severity of disease and predicting the prognosis. Importantly, such knowledge can help clinicians in developing early intervention plans and improve the prognosis of stroke patients.

In the abovementioned context, it is hypothesized that ALS, another disorder characterized by motor dysfunction and CST degeneration, may induce varying degrees of impairment in CST subfibers. In previous studies, researchers have found that there are impairments in CST in ALS patients,^24^ but studies have not specifically assessed which CST subfibers exhibit the most serious damage in ALS. The primary aims of this study are (1) to demonstrate the improved sensitivity and specificity of the NODDI over conventional DTI for detecting CST injury in ALS, (2) to determine using NODDI which CST subfibers show the most severe impairment in ALS, (3) to test the relationship between CST subfiber impairment and ALS disease severity, and (4) to determine the effectiveness of NODDI parameters in CST subfibers as in vivo imaging biomarkers for the diagnosis of ALS.

## 2 MATERIALS AND METHODS

### 2.1 Subjects

We obtained approval for this study from the Research Ethics Committee of Fujian Medical University Union Hospital, China; and all study subjects provided written informed consent before enrollment. A total of 39 sporadic patients with ALS and 50 healthy controls (HC) were enroled in this study. The El Escorial criteria^32^ were used to diagnose ALS, and the revised ALS Functional Rating Scale (ALSFRS‐R) was used to assess disease severity. No significant differences were found between the study groups with respect to age, sex, or years of education (Table 1). The study exclusion criteria included (1) other neuropsychiatric disorders, such as Alzheimer's disease, Parkinson's disease, depression, or epilepsy; (2) taking psychotropic medications; (3) suffering from respiratory failure or another serious disorder like angiocardiopathy or cancer; and (4) contraindication to MRI examination.

**TABLE 1:** Subject demographic and clinical characteristics.

	HC group	ALS group	*p* Value
	(*n* = 50)	(*n* = 39)	
Age (years)	53.8 ± 9.9	53.2 ± 10.4	0.761
Sex (female/male)	20/30	17/22	0.733
Education (years)	8.2 ± 3.8	8.4 ± 4.3	0.808
Site of onset (bulbar/cervical/lumbosacral)	–	6/26/7	–
Diagnostic category (definite/probable)	–	11/28	–
ALSFRS–R score	–	39.1 ± 6.1	–
Disease duration (months)	–	16.3 ± 10.6	–
Disease progression rate	–	0.79 ± 0.74	–

*Note*: The disease progression rate was calculated as (48 − ALSFRS‐R score)/disease duration. “‐” denotes no data available.

Abbreviations: ALS, amyotrophic lateral sclerosis; ALSFRS‐R, revised ALS Functional Rating Scale; HC, healthy control.

### 2.2 MRI data acquisition

We used a 3 T MRI scanner (Prisma; Siemens Medical Systems, Erlangen, Germany) to obtain images. dMRI data were obtained using a multi‐shell spin‐echo echo‐planar imaging sequence, using *b* values = 1000, 2000, and 3000 s/mm^2^, with 30, 30, and 30 unique gradient directions, respectively. Six *b* value = 0 images were also acquired. The other parameters of interest were: repetition time = 4200 ms, echo time = 72 ms, number of averages = 1, slice thickness = 2 mm, the field of view = 216 mm × 216 mm, matrix = 108 × 108, voxel size = 2 mm × 2 mm × 2 mm, flip angle = 90°, 72 axial slices without gap, anterior‐to‐posterior phase direction, and multiband factor = 2. We also collected T1‐weighted (T1W) three‐dimensional magnetization‐prepared rapid gradient echo sagittal images using the following parameters: repetition time = 1610 ms, echo time = 2.25 ms, inversion time = 900 ms, field of view = 224 mm × 224 mm, matrix = 224 × 224, flip angle = 8°, slice thickness = 1.0 mm, and slice number = 176.

### 2.3 Image preprocessing and diffusion metric calculation

The preprocess and modeling of dMRI data were carried out using FSL6.0 (https://fsl.fmrib.ox.ac.uk), SPM12 (https://www.fil.ion.ucl.ac.uk/spm/software/spm12), and MDT v1.2.6 (https://github.com/robbert‐harms/MDT). First, the *b*0 dMRI image was skull‐stripped to generate the brain mask. Then, the dMRI images underwent eddy current and head motion corrections. Next, DTI and NODDI models were estimated based on the corrected dMRI data within the brain mask. For DTI modeling, a standard linear regression model was used to fit the tensor using the “dtifit” function in FSL, and several metrics were calculated based on the tensor, including fractional anisotropy (FA), mean diffusivity (MD), axial diffusivity (AD), and radial diffusivity (RD). Meanwhile, for NODDI modeling, a Powell conjugate‐direction algorithm^33^ was used to fit the model based on MDT, and NDI and ODI values were calculated. Then, the T1W image was coregistered to the subject's *b*0 image, segmented, and non‐linearly normalized into the Montreal Neurological Institute (MNI) space using a Diffeomorphic Anatomical Registration Through Exponentiated Lie Algebra (DARTEL) algorithm.^34^ All diffusion metrics were subsequently written into the MNI space using the DARTEL warp parameters with a 2‐mm cubic resliced voxel. Finally, the normalized metrics were smoothed only for voxel‐based statistics using a Gaussian kernel full width at half‐maximum (FWHM) of 6 × 6 × 6 mm^3^.

### 2.4 CST and its subfiber parcellation

**FIGURE 1:**
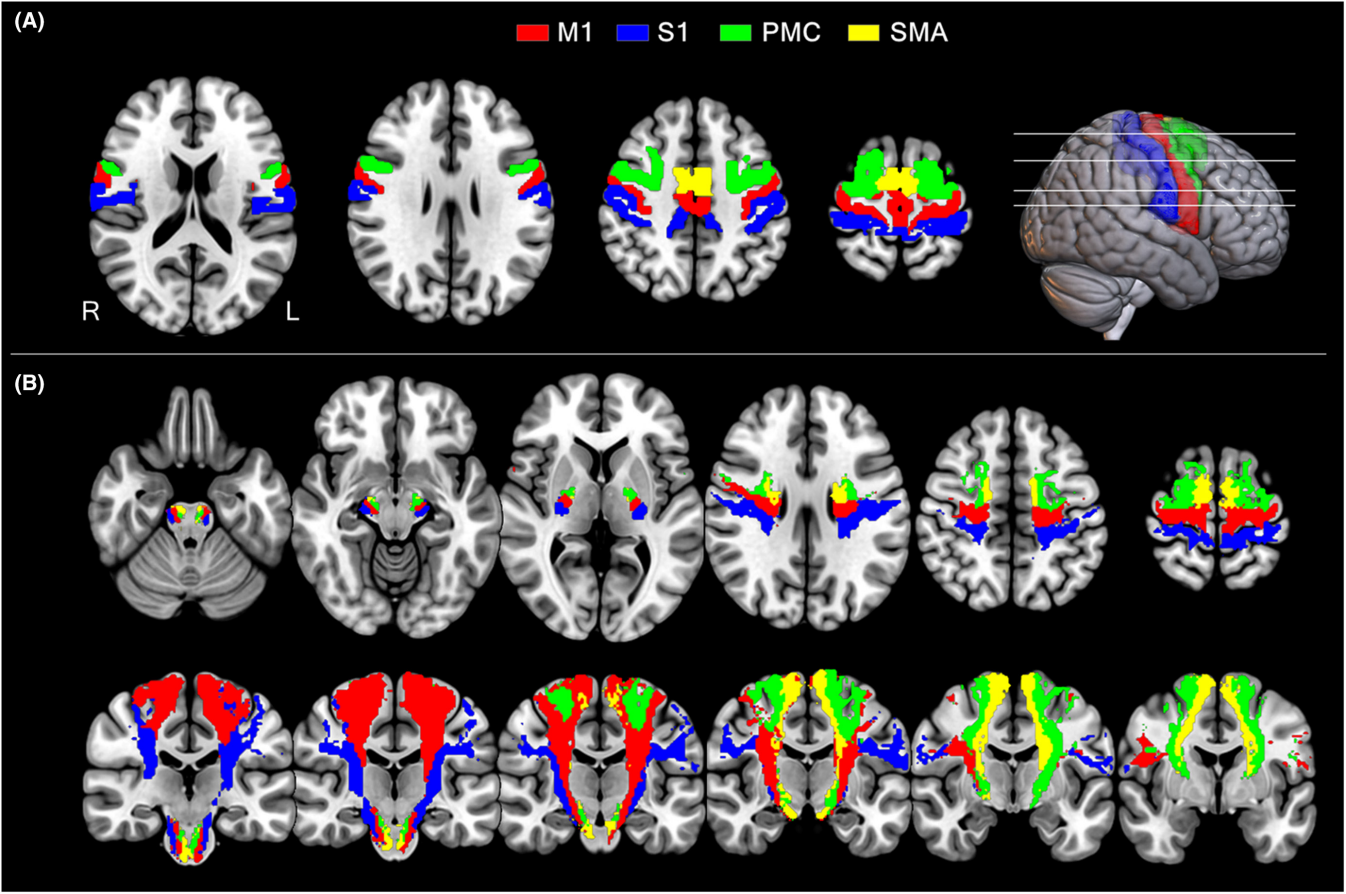
The maximum probability maps of the CST subfibers. (A) Cortical regions of interest for reconstructing CST subfibers, including M1 (red), PMC (green), S1 (blue), and SMA (yellow) fibers. (B) The CST subfibers (red, M1 fibers; green, PMC fibers; blue, S1 fibers; and yellow, SMA fibers) are reconstructed with 50% probability. CST, corticospinal tract; L, left; M1, primary motor area; PMC, premotor cortex; R, right; S1, primary sensory area; SMA, supplementary motor area.

In terms of anatomy, the origins of CST fibers include four cortical areas, namely, M1, PMC, SMA, and S1.^26^, ^27^, ^35^ In this study, the bilateral CSTs and their subfibers (i.e., M1, PMC, SMA, and S1 fibers) were retrieved from a recent study whose authors reconstructed the fine maps of CSTs and their subfibers using data from 50 healthy adults from the Human Connectome Project (http://www.humanconnectomeproject.org/).^31^ A maximum probability map was created with a population probability of ≥50% for each CST subfiber originating from the M1, PMC, S1, and SMA (Figure 1). Besides, a mask for the whole CST for each side with a population probability of >50% was also generated. Finally, the averaged diffusion metrics within each CST and CST subfiber for each subject were calculated from the unsmoothed normalized diffusion maps for region of interest (ROI)‐wise statistics. These parcellation maps were also used as masks for voxel‐wise statistics.

### 2.5 Statistical analysis

The Kolmogorov–Smirnov test was applied to examine the normality. Then, we used generalized linear modeling (GLM) to compare inter‐group differences in averaged diffusion metrics for each ROI of CST fiber, adopting age, sex, and year of education as the nuisance covariates (*p* < 0.05 with Bonferroni correction). Cohen's *D* value was calculated to quantify the effect size (ES) for each comparison. Besides, to identify the specific location of the changed diffusion metric in each fiber, we also carried out voxel‐wise intergroup comparisons using GLM while controlling for age, sex, and year of education (*p* < 0.05, voxel‐wise family‐wise error [FWE] correction). The percentage of voxels with diffusion metric abnormalities was quantified for each fiber. The Spearman's correlation was used to examine the association between NODDI metrics of each CST subfiber and disease severity as reflected by the ALSFRS‐R score (*p* < 0.05 with Bonferroni correction). Finally, to evaluate the potential of the NODDI metrics of CST subfibers in diagnosing ALS, we carried out logistic regression with NDI values of each CST subfiber (and their combination) as features and group ID (1 = ALS patients, 0 = HCs) as labels. The area under the receiver operating characteristic (ROC) curve (AUC) was used to estimate the predicted performance, and a paired Delong's test was used to compare the AUC values between each pair of models (*p* < 0.05 was considered statistically significant).

## 3 RESULTS

### 3.1 ROI‐wise intergroup differences in diffusion metrics

**FIGURE 2:**
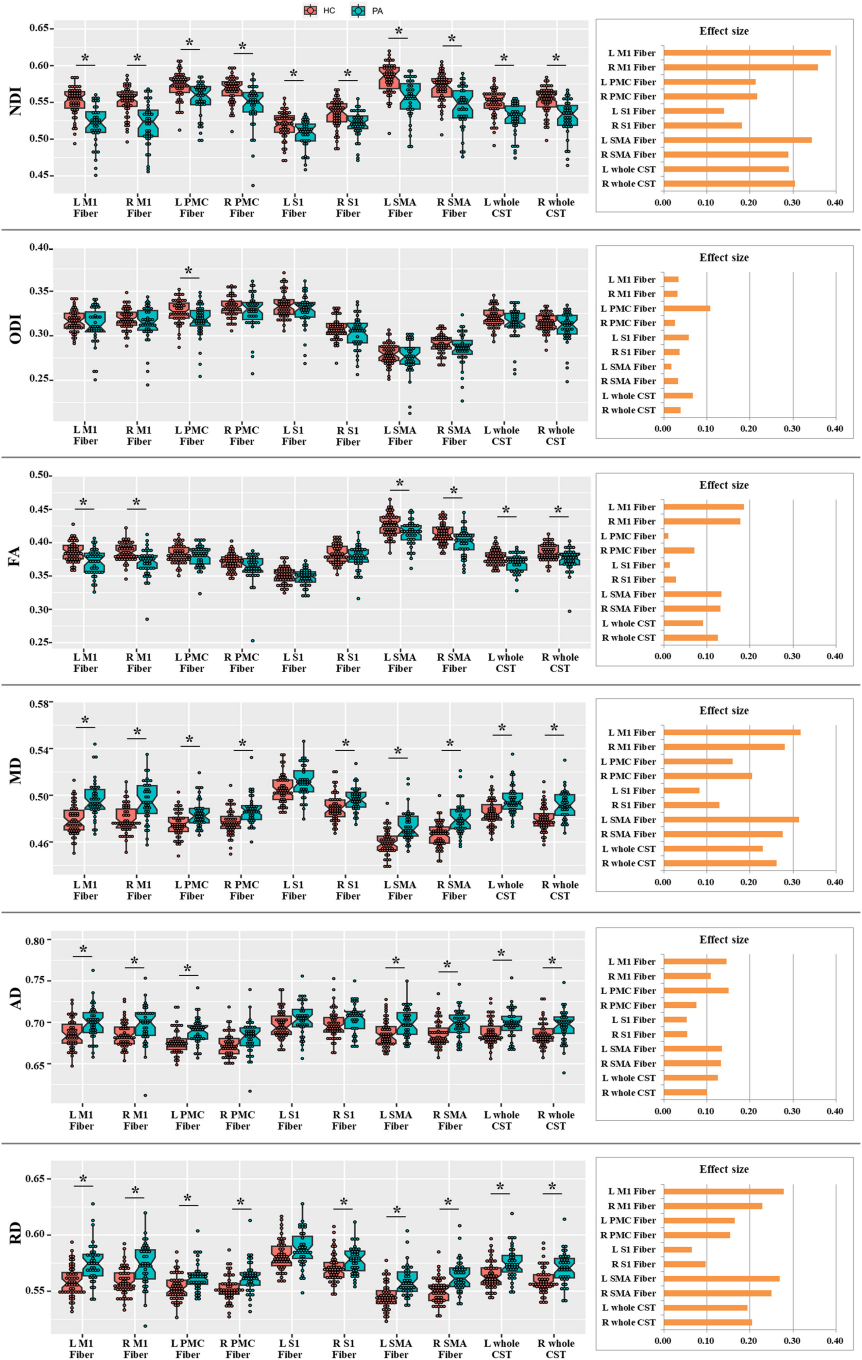
The between‐group differences in the averaged values of diffusion parameters along the CST subfibers. The unit of MD, AD, and RD parameters is “×10^−3^ mm^2^/s”. “*” denotes the corrected *p* value < 0.05.

The between‐group differences in the diffusion metrics among the CST subfibers are depicted in Figure 2. Compared with HCs, patients with ALS showed decreased NDI values for all CST subfibers and bilateral whole CSTs (*p* < 0.05, Bonferroni correction; averaged ES across fibers = 0.272 ± 0.081); however, patients with ALS only exhibited a tiny decrease in ODI in left PMC fiber (averaged ES across fibers = 0.045 ± 0.026). In addition, FA reductions in several CST subfibers (including bilateral M1 and SMA fibers) and bilateral whole CSTs were observed in patients with ALS (averaged ES across fibers = 0.097 ± 0.065). MD (averaged ES across fibers = 0.226 ± 0.080), AD (averaged ES across fibers = 0.108 ± 0.036), and RD (averaged ES across fibers = 0.191 ± 0.071) increases in the majority of CST subfibers and bilateral whole CSTs were also found in patients with ALS, although they displayed relatively lower ES values compared with the NDI.

Among the CST subfibers, bilateral M1 and SMA subfibers had a greater effect size of microstructural changes for NDI (averaged ES across bilateral M1 fibers = 0.373 ± 0.020; averaged ES across bilateral SMA fibers = 0.316 ± 0.039) relative to other CST subfibers and bilateral whole CSTs. A similar pattern was also observed for DTI metrics (including FA, MD, and RD). In addition, across the diffusion metrics, the average ES in bilateral M1 fibers (ES = 0.210 ± 0.118) was larger than that in SMA fibers (ES = 0.190 ± 0.110), PMC fibers (ES = 0.130 ± 0.070), S1 fibers (ES = 0.080 ± 0.050), and whole CSTs (ES = 0.170 ± 0.090), further indicating that the diffusion measurement of M1 fibers was much more sensitive in detecting microstructural abnormalities compared with that of other subfibers and the whole CST.

### 3.2 Voxel‐wise intergroup differences in diffusion metrics

The voxel‐wise differences in distinct diffusion parameters among the CST subfibers are presented in Figures [Link], [Link]. Decreases in NDI and ODI were found in patients with ALS (*p* < 0.05, voxel‐wise FWE correction). Along each of the seven CST subfibers (including bilateral M1, PMC, and S1 fibers and left SMA fibers), the voxel‐wise analysis for NDI revealed the greatest extent of between‐group difference compared with the analyses for the other diffusion metrics (*p* < 0.05, voxel‐wise FWE correction). Meanwhile, a lesser extent of ODI difference was observed along bilateral PMC, S1, and SMA fibers. In addition, the DTI parameter differences (as reflected by decreased FA and increased MD, AD, and RD values in ALS) were found among the seven CST subfibers, including bilateral M1, PMC, and S1 fibers and left SMA fiber; but the extent was smaller than that seen with the NDI difference.

**FIGURE 3:**
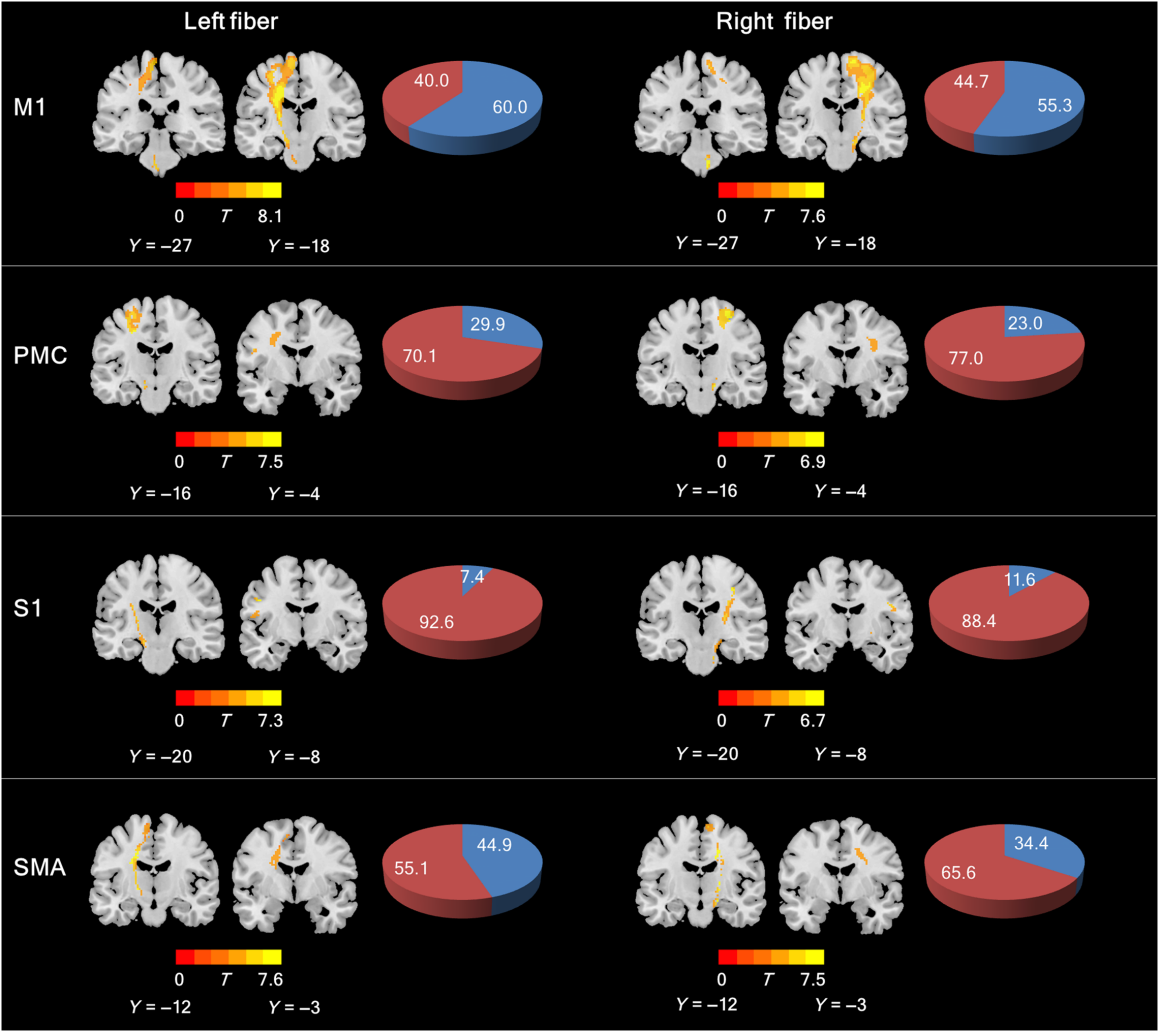
The regions with between‐group differences in the NDI parameter. The pie chart indicates the extent of the abnormalities detected by NDI measurement. The detected (blue) and non‐detected (red) extents are each expressed as the percentage of the total volume of each CST subfiber.

Figure 3 shows the regions with NDI reduction along all CST subfibers in patients with ALS. Of note, the extent of NDI reduction was greatest in bilateral M1 fibers, followed by, in the order, bilateral SMA, PMC, and S1 fibers. A similar pattern also existed for DTI metrics (Figures [Link], [Link]).

### 3.3 Result of correlation analysis

**FIGURE 4:**
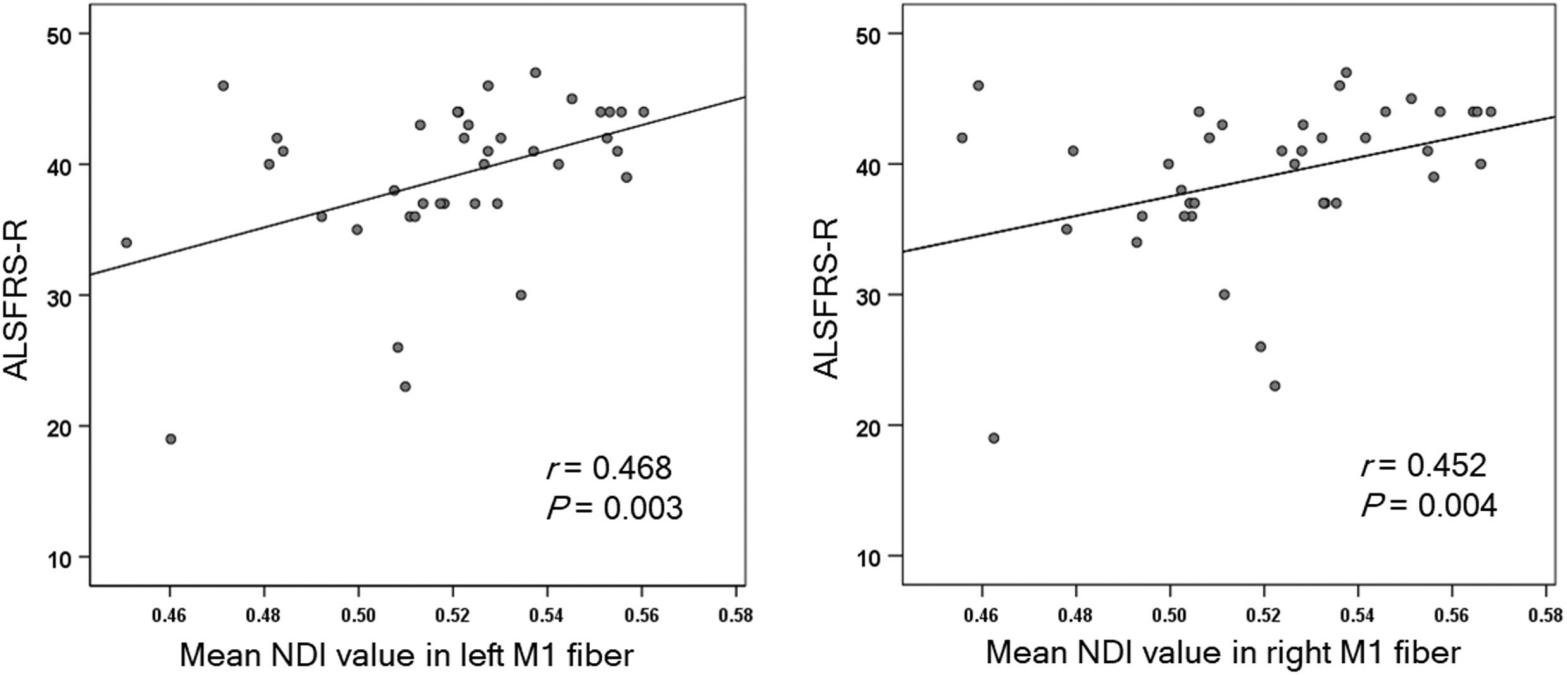
The correlation between CST impairment assessed by the NDI parameter and disease severity assessed by ALSFRS‐R. The *p* value displayed is < 0.05 after Bonferroni correction.

The mean NDI value in bilateral M1 fibers was significantly positively correlated with the ALSFRS‐R score (*p* < 0.05, Bonferroni correction; Figure 4). No significant correlation was found between the NDI values of other subfibers and the whole CST and ALSFRS‐R score.

### 3.4 Results of classification analysis

Among the majority of CST fibers, the analyses based on NDI showed better classification performance compared with those based on other diffusion metrics (Table S1). Specifically, along M1 fiber, the NDI index yielded the best classification performance (accuracy = 77.5%, *p* < 0.001) followed by, in the order, MD (accuracy = 74.2%, *p* < 0.001), FA (accuracy = 73.0%, *p* < 0.001), RD (accuracy = 71.9%, *p* < 0.001), AD (accuracy = 65.2%, *p* = 0.003), and ODI (accuracy = 57.3%, *p* = 0.285). A similar pattern was observed along S1 fiber, SMA fiber, and whole CST.

Among the distinct CST subfibers, M1 fiber exhibited the more important role in classification analyses because its NDI provided a higher classification accuracy (77.5%) relative to the NDI of other CST subfibers. Moreover, a similar pattern was found for DTI metrics (including MD, FA, and RD). Of note, the diffusion index along several CST subfibers, especially M1, showed a promising classification accuracy (77.5%), which was higher than that obtained in analyses performed using the diffusion metrics of the whole CST as an index.

### 3.5 Results of ROC curve analysis

**FIGURE 5:**
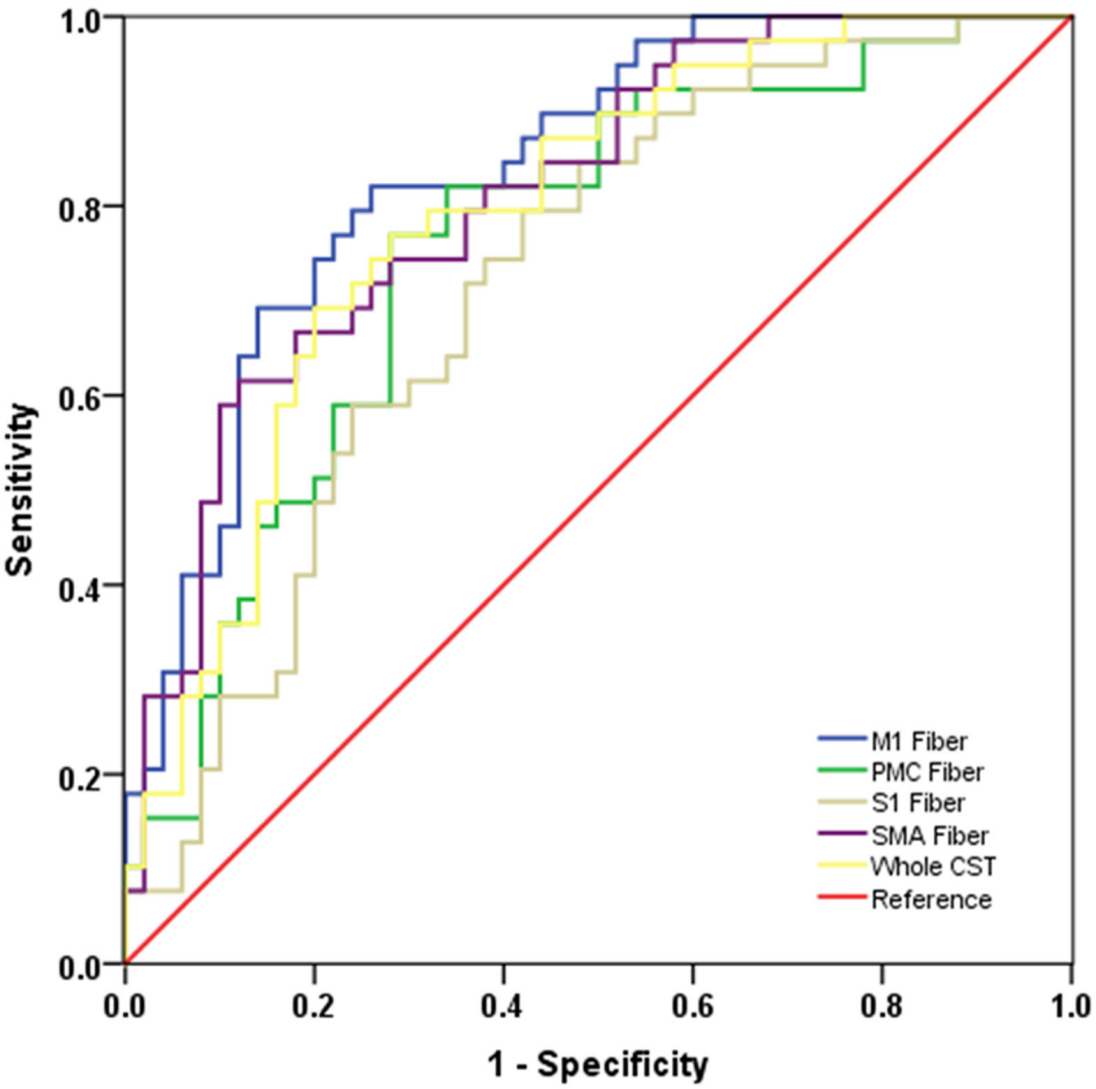
The results of the ROC analyses using the NDI parameter along the whole CST and CST subfibers as an index.

For each of the CST subfibers, the ROC curve analysis based on the NDI metric exhibited a greater AUC for the diagnosis of ALS compared with that based on other diffusion metrics (Table S2). As Figure 5 shows, the ROC curve analysis performed using the NDI metric in M1, SMA, PMC, and S1 fibers yielded a moderate diagnostic power, and the corresponding AUC was, in order of high to low, 0.839, 0.811, 0.758, and 0.719 (all *p* < 0.001). Statistically, the NDI of M1 fiber yielded a higher AUC than that of PMC fiber (*z* = 2.294, *p* = 0.011), S1 fiber (*z* = 3.065, *p* = 0.001), and the whole CST (*z* = 1.919, *p* = 0.027). Besides, the NDI of SMA fiber had a higher AUC than that of PMC (*z* = 2.030, *p* = 0.021) and S1 (*z* = 2.402, *p* = 0.008) fibers.

## 4 DISCUSSION

In the current study, we adopted NODDI for the first time to investigate the various degrees of microstructural impairment in CST subfibers with different origins in patients with ALS. Our main findings can be summarized as follows: (1) NODDI provided more sensitive metrics than DTI for detecting CST subfiber impairment; (2) patients with ALS showed microstructural impairments in CST subfibers (especially M1 and SMA fibers), which was reflected by NDI, ODI, and FA reductions and MD, AD, and RD increases; (3) M1 fiber microstructural impairment correlates with disease severity in ALS; (4) among CST subfibers, M1 fiber showed the greatest degree of microstructural impairment in ALS; (5) compared with other CST subfibers, M1 fiber exhibited the most important role in identifying ALS; and (6) compared with the whole CST analysis, CST subfiber analysis improved the accuracy for diagnosing ALS. To summarize, the CST subfiber analysis based on NODDI could contribute to identifying more sensitive biomarkers, which would be helpful for diagnosing and assessing disease severity in ALS.

Research suggests a neuropathological substrate underlying WM microstructural impairment in ALS is associated with axonal degeneration.^36^, ^37^ Consistently, we detected widespread NDI decrease in CST fibers, suggesting that the reduction of axonal density is the conspicuous pathological process in ALS.^17^, ^20^ In addition, our finding of NDI reduction in ALS is consistent with the results of previous diffusion MRI studies demonstrating that the loss of motor neuron axons is a core feature of the neurodegenerative process in ALS.^11^, ^38^ It has been found that the axonal loss in ALS contributes to the mutation of genes encoding cytoskeletal proteins, which are indispensable for the maintenance of axonal transport and integrity, and the dysfunction of astrocytes and oligodendrocytes, which regulate myelination to maintain axonal function.^39^, ^40^, ^41^, ^42^, ^43^, ^44^ In addition, a relatively rare reduction of ODI was observed along CST fibers in patients with ALS in accordance with previous NODDI research that demonstrated the small extent of the WM region with ODI reduction in ALS.^24^, ^45^ This ODI reduction may be explained as a result of the decreased crossing of fibers, leaving behind a more aligned organization in the relevant WM regions.^17^, ^24^

In a previous neuroimaging study, a reduction in the volume of blood flow has been found in bilateral SMA fibers in patients with ALS, yet the blood flow remained steady in other CST subfibers,^46^ suggesting the non‐uniformity of impairment degree among CST subfibers. Consistently, we found that CST subfibers exhibited distinct degrees of microstructural impairment (e.g., as reflected by NDI reduction) in ALS. One of the earliest pathological hallmarks in ALS is the degeneration of the Betz cell in the M1 cortex,^47^, ^48^ which may help to explain the greatest degree of axonal density reduction observed in M1 fiber. M1 and its fibers play a crucial role in the execution of voluntary movements,^29^, ^30^ which suggests that altered microstructures in these regions may contribute to the disability of movement execution that has been well reported in ALS.^49^ In addition, given that the SMA is responsible for the coordination of internally generated movement,^29^, ^30^ the impaired microstructures in the SMA and its fibers may induce dysfunction in coordinating complicated actions seen in ALS.^50^ Given that the PMC and S1 cortex are, respectively, involved in visually guided movements and descending control of sensory afferent input generated by movements,^29^, ^30^ microstructural impairment in their fibers may be associated with the deficits in relevant motor functions seen in ALS.^51^, ^52^ Taken together, the impairment of CST subfibers with different origins could affect the corresponding domain of movement function in ALS; moreover, CST subfiber analysis could effectively overcome the generality deficiency in the whole‐CST analysis method and could more comprehensively evaluate the ALS‐related degeneration in each subfiber, which might improve the diagnosis of ALS.

Our findings suggest that NODDI could ensure higher sensitivity and greater tissue specificity than DTI in detecting CST abnormalities, concurring with previous studies in which voxel‐based^24^ and atlas‐based^45^ analyses consistently demonstrated the added sensitivity and specificity of NODDI over DTI in identifying ALS‐related impairments in many WM areas. The increased sensitivity and specificity of NODDI is associated with its capability to disentangle the contributions of axonal density and fiber orientation to microscopic changes, which are both represented by FA change.^53^ This advantage of NODDI over DTI is associated with the three‐compartment tissue model applied in NODDI, which allows for the interrogation of both intra‐ and extracellular properties of WM tissue to enable the differentiation of two key aspects of axonal pathology, which are the packing density of axons (reflected by NDI) and the spatial organization of the axons (reflected by ODI).^17^

Several limitations in the current study must be acknowledged. First, the small sample size used in the current study could have limited its statistical power. Second, as reported,^2^ WM microstructural impairments can differ among the distinct phenotypes of ALS. As such, a larger population is required to disentangle the influences of ALS heterogeneity (e.g., genetic origin, site of onset, or disease‐progression rate) on the NDI and ODI. Third, this study did not include a longitudinal investigation to observe microstructural impairments along CST subfibers over time, thereby preventing us from gaining a direct understanding of NDI and ODI alterations associated with ALS disease progression.

In conclusion, the microstructural impairment of CST subfibers (especially those originating from M1) is the key feature of ALS. NODDI can offer greater sensitivity and better tissue specificity over DTI in detecting microstructural impairments among CST subfibers in ALS. The combination of NODDI and CST subfibers analysis may improve diagnosing performance for ALS.

## FUNDING INFORMATION

This research was supported by grant from the Fujian Provincial Health Technology Project (no. 2022QNA022).

## CONFLICT OF INTEREST STATEMENT

The authors declare that they do not have any conflicts of interest.

## Supporting information


Figure S1

Figure S2

Figure S3

Figure S4

Appendix S1

## Data Availability

The data that support the findings of this study are available from the corresponding author upon reasonable request.
