# Supplementary figures and images for: Corticospinal fibers with different origins impair in amyotrophic lateral sclerosis: A neurite orientation dispersion and density imaging study
Source: CNS Neurosci Ther. 2023 May 19;29(11):3406–15. doi: 10.1111/cns.14270 (PMC10580332; doi:10.1111/cns.14270)

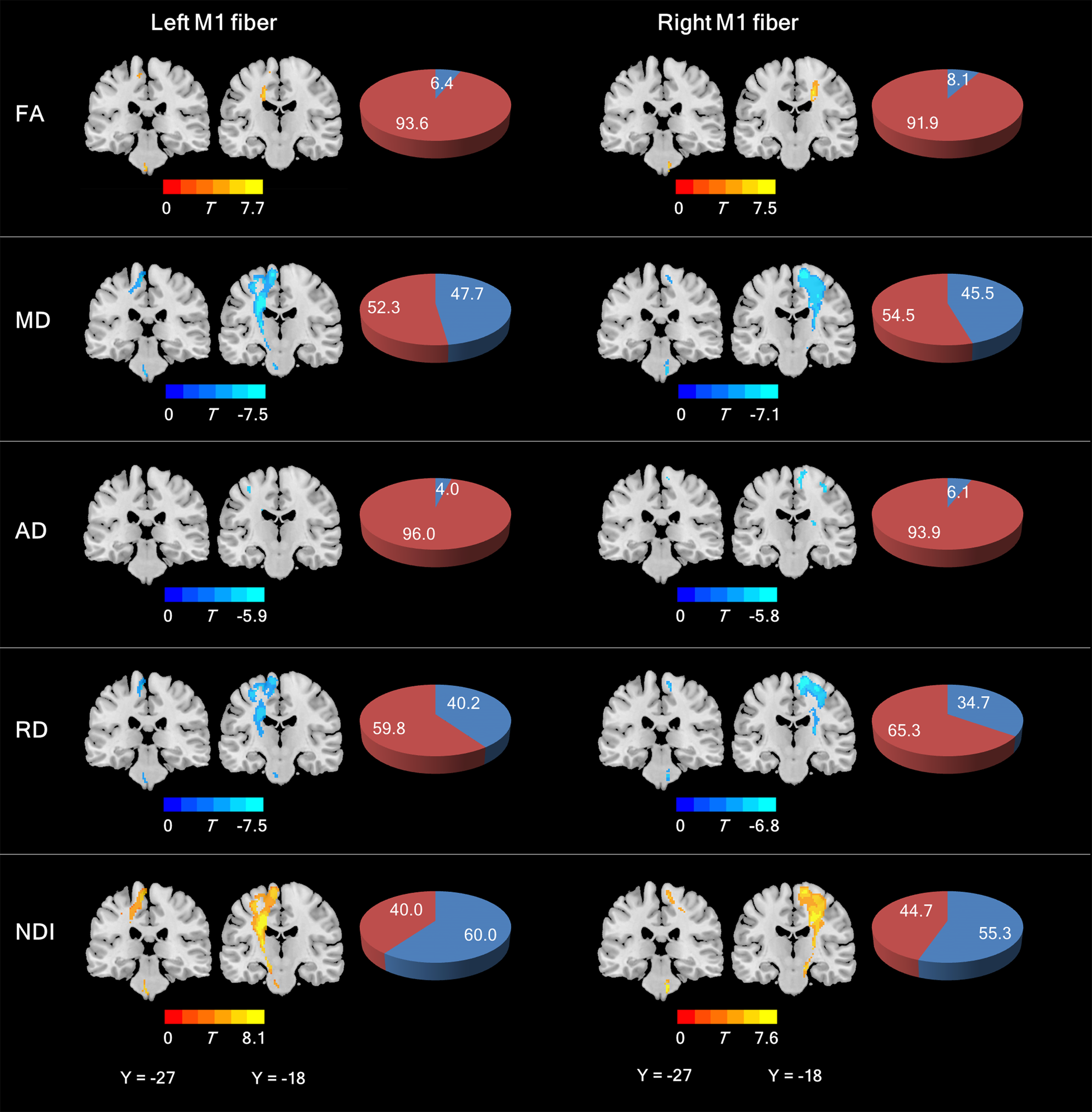

Supplement: Supplementary file 1 — Figure S1 [file CNS-29-3406-s001.tif]

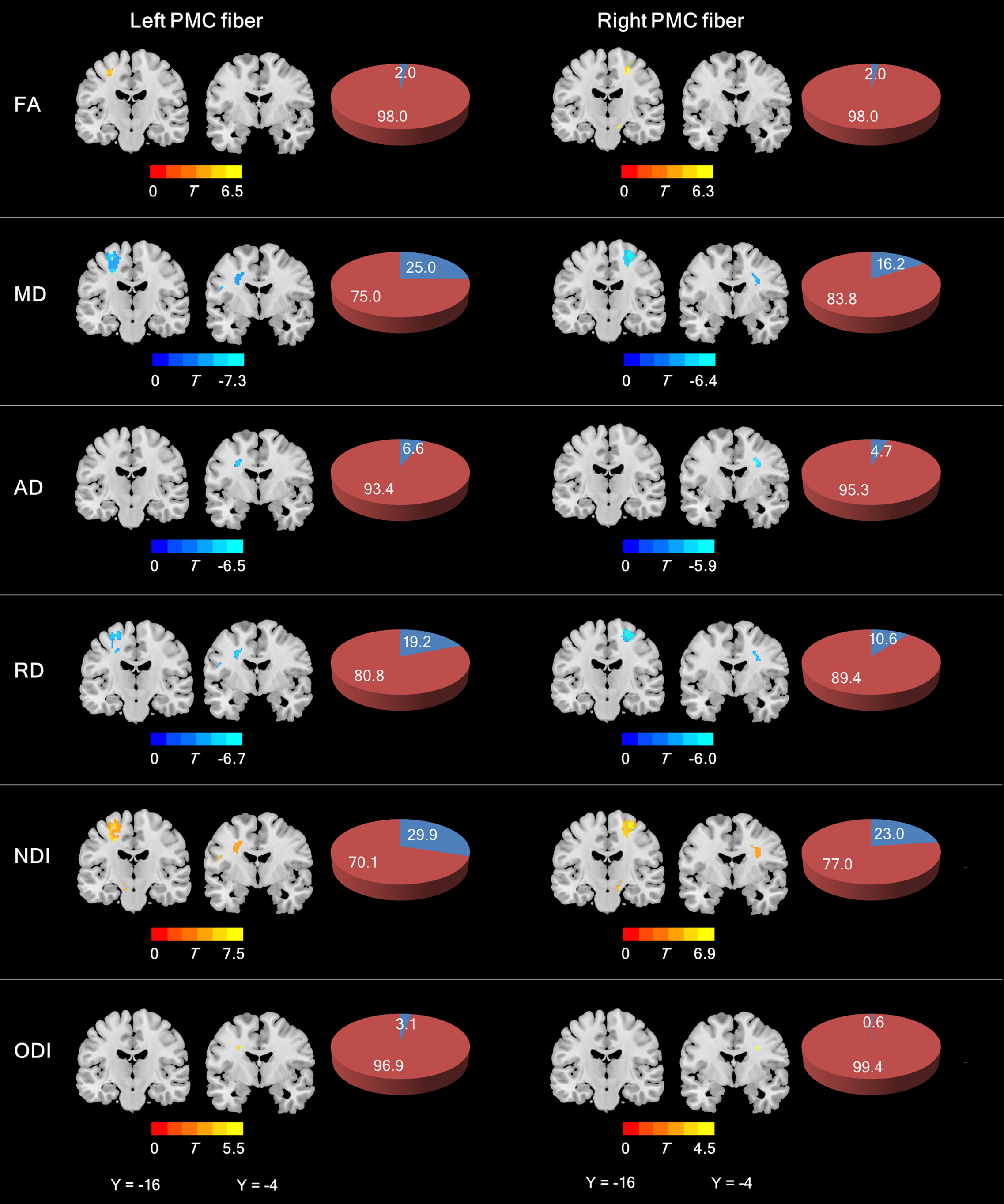

Supplement: Supplementary file 2 — Figure S2 [file CNS-29-3406-s005.tif]

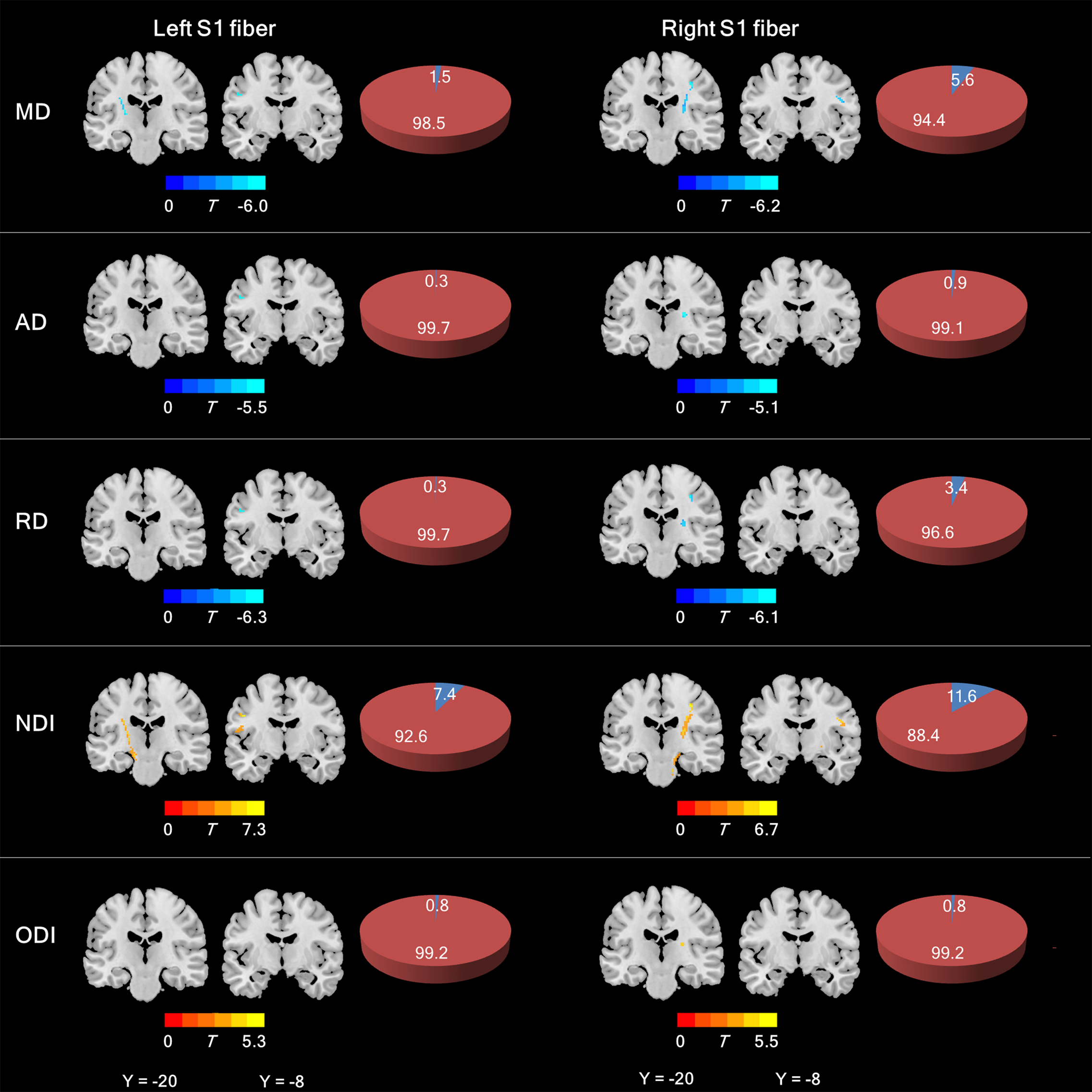

Supplement: Supplementary file 3 — Figure S3 [file CNS-29-3406-s003.tif]

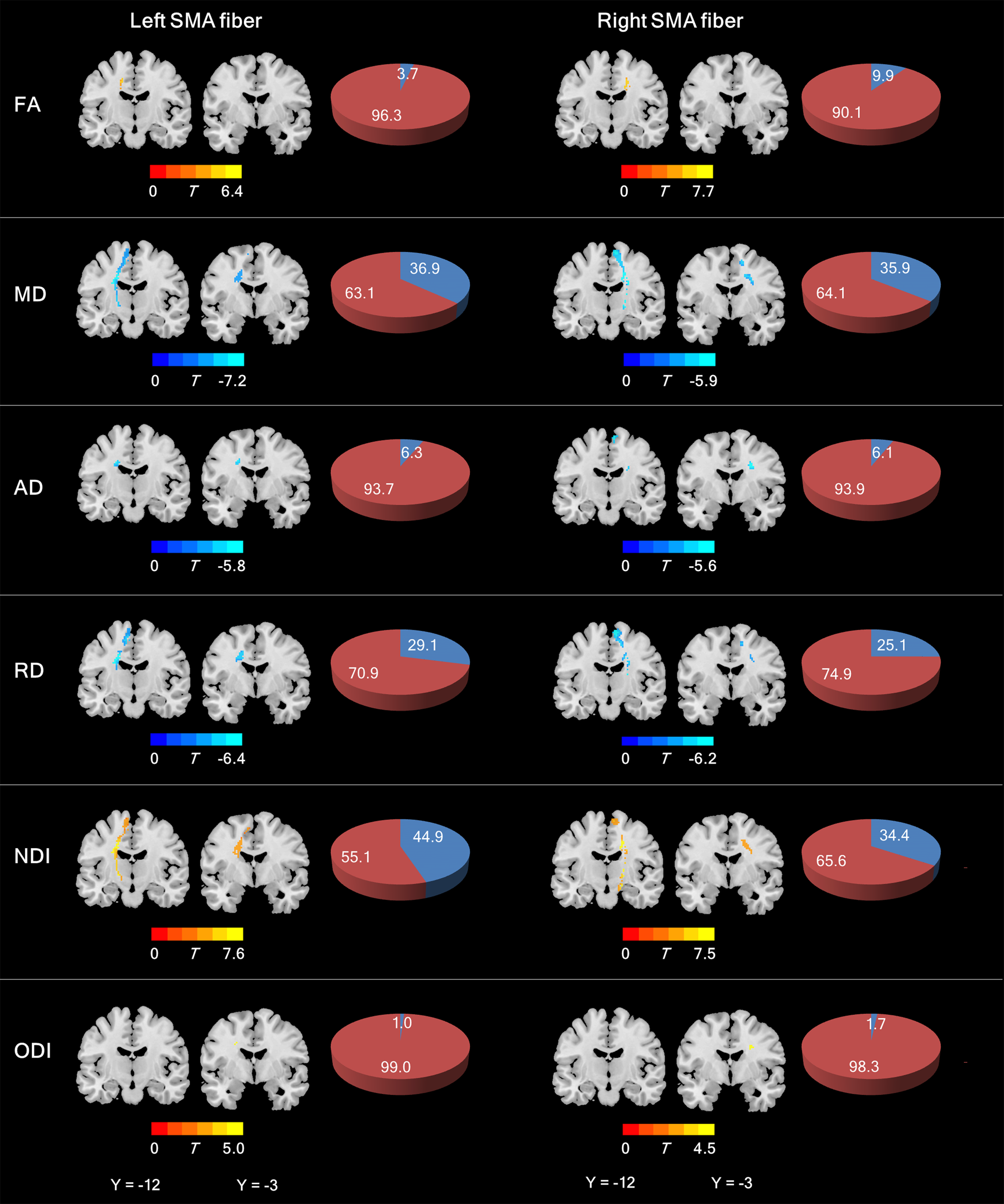

Supplement: Supplementary file 4 — Figure S4 [file CNS-29-3406-s002.tif]
