# Supplementary material for: Corticospinal fibers with different origins impair in amyotrophic lateral sclerosis: A neurite orientation dispersion and density imaging study
Source: CNS Neurosci Ther. 2023 May 19;29(11):3406–15. doi: 10.1111/cns.14270 (PMC10580332; doi:10.1111/cns.14270)
Supplement: Supplementary file 5 — Appendix S1 [file CNS-29-3406-s004.docx]

**Supplementary Figure 1.** The between-group differences in the distinct diffusion parameters along the M1 fiber. The pie chart indicates the extent of the abnormalities detected by diffusion parameters. The detected (blue) and non-detected (red) extents are expressed as the percentage of the total volume of each CST subfiber. *Abbreviations:* FA, fractional anisotropy; MD, mean diffusivity; AD, axial diffusivity; RD, radial diffusivity; NDI, neurite density index.

**Supplementary Figure 2.** The between-group differences in the distinct diffusion parameters along the PMC fiber. The pie chart indicates the extent of the abnormalities detected by diffusion parameters. The detected (blue) and non-detected (red) extents are expressed as the percentage of the total volume of each CST subfiber. *Abbreviations:* FA, fractional anisotropy; MD, mean diffusivity; AD, axial diffusivity; RD, radial diffusivity; NDI, neurite density index; ODI, orientation dispersion index.

**Supplementary Figure 3.** The between-group differences in the distinct diffusion parameters among the S1 fibers. The pie chart indicates the extent of the abnormalities detected by diffusion parameters. The detected (blue) and non-detected (red) extents are expressed as the percentage of the total volume of each CST subfiber. *Abbreviations:* MD, mean diffusivity; AD, axial diffusivity; RD, radial diffusivity; NDI, neurite density index; ODI, orientation dispersion index.

**Supplementary Figure 4.** The between-group differences in the distinct diffusion parameters along the SMA fiber. The pie chart indicates the extent of the abnormalities detected by diffusion parameters. The detected (blue) and non-detected (red) extents are expressed as the percentage of the total volume of each CST subfiber. *Abbreviations:* FA, fractional anisotropy; MD, mean diffusivity; AD, axial diffusivity; RD, radial diffusivity; NDI, neurite density index; ODI, orientation dispersion index.

Supplementary Table 1. The results of classification analyses using the diffusion parameters along the whole CST and the CST subfibers as the index

|  | Fibers | Sensitivity (%) | Specificity (%) | Accuracy (%) | *χ^2^* value | *P* value |
| --- | --- | --- | --- | --- | --- | --- |
| FA | M1 | 53.8 | 88.0 | 73.0 | 19.35 | <0.001 |
|  | PMC | 43.6 | 82.0 | 65.2 | 8.43 | 0.015 |
|  | S1 | 20.5 | 86.0 | 57.3 | 2.75 | 0.252 |
|  | SMA | 46.2 | 72.0 | 60.7 | 13.17 | 0.001 |
|  | Whole CST | 51.3 | 80.0 | 67.4 | 12.79 | 0.002 |
| MD | M1 | 66.7 | 80.0 | 74.2 | 28.08 | <0.001 |
|  | PMC | 48.7 | 76.0 | 64.0 | 13.94 | <0.001 |
|  | S1 | 28.2 | 80.0 | 57.3 | 5.39 | 0.020 |
|  | SMA | 61.5 | 80.0 | 71.9 | 28.47 | <0.001 |
|  | Whole CST | 59.0 | 76.0 | 68.5 | 18.33 | <0.001 |
| AD | M1 | 53.8 | 74.0 | 65.2 | 11.39 | 0.003 |
|  | PMC | 53.8 | 78.0 | 67.4 | 12.98 | <0.001 |
|  | S1 | 30.8 | 80.0 | 58.4 | 3.21 | 0.073 |
|  | SMA | 48.7 | 78.0 | 65.2 | 11.43 | 0.001 |
|  | Whole CST | 51.3 | 78.0 | 66.3 | 9.34 | 0.002 |
| RD | M1 | 64.1 | 78.0 | 71.9 | 23.38 | <0.001 |
|  | PMC | 59.0 | 80.0 | 70.8 | 14.19 | <0.001 |
|  | S1 | 28.2 | 80.0 | 57.3 | 3.96 | 0.047 |
|  | SMA | 53.8 | 82.0 | 69.7 | 23.53 | <0.001 |
|  | Whole CST | 51.3 | 78.0 | 66.3 | 14.63 | <0.001 |
| NDI | M1 | 69.2 | 84.0 | 77.5 | 34.00 | <0.001 |
|  | PMC | 51.3 | 78.0 | 66.3 | 18.39 | <0.001 |
|  | S1 | 51.3 | 78.0 | 66.3 | 12.35 | 0.002 |
|  | SMA | 66.7 | 80.0 | 74.2 | 27.01 | <0.001 |
|  | Whole CST | 61.5 | 82.0 | 73.0 | 23.52 | <0.001 |
| ODI | M1 | 17.9 | 88.0 | 57.3 | 2.50 | 0.285 |
|  | PMC | 53.8 | 76.0 | 66.3 | 10.36 | 0.006 |
|  | S1 | 25.6 | 78.0 | 55.1 | 3.49 | 0.175 |
|  | SMA | 25.6 | 84.0 | 58.4 | 2.82 | 0.244 |
|  | Whole CST | 33.3 | 80.0 | 59.6 | 5.39 | 0.067 |

*Abbreviations:* HC, healthy control; ALS, amyotrophic lateral sclerosis; FA, fractional anisotropy; MD, mean diffusivity; AD, axial diffusivity; RD, radial diffusivity; NDI, neurite density index; ODI, orientation dispersion index; ISO, isotropic compartment.

Supplementary Table 2. Results of the ROC analyses using the diffusion parameters along the whole CST and the CST subfibers as the index

| Diffusion parameter | M1 fiber | | PMC fiber | | S1 fiber | | SMA fiber | | Whole CST | |
| --- | --- | --- | --- | --- | --- | --- | --- | --- | --- | --- |
|  | AUC | *P* value | AUC | *P* value | AUC | *P* value | AUC | *P* value | AUC | *P* value |
| FA | 0.735 | **<0.001** | 0.666 | **0.008** | 0.574 | 0.231 | 0.698 | **0.001** | 0.701 | **0.001** |
| MD | 0.805 | **<0.001** | 0.711 | **0.001** | 0.644 | 0.021 | 0.814 | **<0.001** | 0.758 | **<0.001** |
| AD | 0.702 | **0.001** | 0.736 | **<0.001** | 0.619 | 0.054 | 0.707 | **0.001** | 0.688 | **0.002** |
| RD | 0.787 | **<0.001** | 0.722 | **<0.001** | 0.642 | 0.022 | 0.796 | **<0.001** | 0.739 | **<0.001** |
| NDI | 0.839 | **<0.001** | 0.758 | **<0.001** | 0.719 | **<0.001** | 0.811 | **<0.001** | 0.791 | **<0.001** |
| ODI | 0.572 | 0.244 | 0.693 | **0.002** | 0.570 | 0.257 | 0.586 | 0.165 | 0.613 | 0.069 |

*Abbreviations:* HC, healthy control; ALS, amyotrophic lateral sclerosis; FA, fractional anisotropy; MD, mean diffusivity; AD, axial diffusivity; RD, radial diffusivity; NDI, neurite density index; ODI, orientation dispersion index; ISO, isotropic compartment. The numbers in bold denote *P* < 0.05 after Bonferroni correction.
